# Musculoskeletal injuries in British Army recruits: a prospective study of diagnosis-specific incidence and rehabilitation times

**DOI:** 10.1186/s12891-015-0558-6

**Published:** 2015-05-04

**Authors:** Jagannath Sharma, Julie P Greeves, Mark Byers, Alexander N Bennett, Iain R Spears

**Affiliations:** Medical Centre Defence Primary Healthcare, Infantry Training Centre Catterick Garrison, DL9 3PS, North Yorkshire, UK; Department of Occupational Medicine, HQ Army Recruiting and Training Division, Trenchard Lines, SN9 6BE Upavon, Wilts, UK; Academic Department of Military Rehabilitation DMRC Headley Court, KT18 6JW, Surrey, UK; School of Social Sciences and Law, Teesside University, TS1 3BA, Middlesbrough, UK

**Keywords:** Military training, Training load, Overuse injury, Rehabilitation

## Abstract

**Background:**

Musculoskeletal injuries during initial military training are a significant medical problem facing military organisations globally. In order to develop an injury management programme, this study aims to quantify the incidence and rehabilitation times for injury specific diagnoses.

**Methods:**

This was a prospective follow-up study of musculoskeletal injuries in 6608 British Army recruits during a 26-week initial military training programme over a 2-year period. Incidence and rehabilitation times for injury specific diagnoses were recorded and analysed.

**Results:**

During the study period the overall incidence of musculoskeletal injuries was 48.6%, and the most common diagnosis was iliotibial band syndrome (6.2%). A significant proportion of the injuries occurred during the first 11 weeks of the programme. The longest rehabilitation times were for stress fractures of the femur, calcaneus and tibia (116 ± 17 days, 92 ± 12 days, and 85 ± 11 days, respectively). The combination of high incidence and lengthy rehabilitation indicates that medial tibial stress syndrome had the greatest impact on training, accounting for almost 20% of all days spent in rehabilitation.

**Conclusion:**

When setting prevention priorities consideration should be given to both the incidence of specific injury diagnoses and their associated time to recovery.

## Background

The British Army provides several intensive training courses to prepare new recruits for combat. The Combat Infantryman’s Course (CIC) at the Infantry Training Centre Catterick (ITC(C)) (UK) is undertaken by more than 3500 recruits each year and lasts for a minimum of 26 weeks. The CIC is considered to be the most physically demanding of all British Army initial military training courses [1] and includes training to improve aerobic fitness, muscle endurance and strength [2] through running, resistance training, battle training and loaded marches [3]. For many recruits, the nature and volume of the physical load is much higher than they have previously experienced [4,5] and it is widely believed that failure to adapt to the large and rapid rise in load increases the risk of musculoskeletal injury [6-8]. The incidence of injuries ranges from 20% to 59% [9-12] and the medical discharge rate at the (ITC(C)) has reached over 8% due, primarily, to musculoskeletal injuries [3]. The injuries have negative impact on morbidity, training time, resources and manning [5,13]. The problem is not unique and occurs across many military training establishments worldwide [14].

There have been several investigations designed to reduce the risk of musculoskeletal injuries during initial military training, however, these interventions have mostly been ineffective [15,16]. The lack of effect may be due to the generic nature of these interventions, which are designed to target all rather than the specific injuries most relevant to the organisation. In contrast, more successful interventions have targeted the mechanisms of specific injuries [17,18] and reported significant beneficial effects on incidence and with no harmful effects on other non-targeted injuries. While such positive findings provide some evidence that a targeted approach to injury prevention could form part of a strategy for initial military training, it should be noted that this approach requires robust epidemiological data from the population in which the intervention is planned [19]. More precisely, there is a need to establish which types of injuries should be targeted and this, in turn, requires that the relative impact of injury specific diagnosis can be quantified in terms of parameters that are meaningful to the organisation.

Despite the high prevalence of musculoskeletal injury there is a shortage of epidemiological data for injuries during initial military training in British Army populations [13,14]. The impact of injury specific diagnoses on a population is an important parameter in injury epidemiology [19]. Many studies have reported the incidence of injury as a primary variable but it is suggested that rehabilitation times associated with these injuries could also be important. Specifically, a lengthy rehabilitation period is costly in terms of resources (e.g. medical costs), lost training days and could lead to a reduction in combat effectiveness [3,13]. Unfortunately, rehabilitation times are less frequently reported but will likely depend upon on a range of factors such as levels of fitness and institutional differences in treatment protocols. The aim of this study was to quantify incidence and rehabilitation times for injury specific diagnoses during initial military training in a large training centre as a foundation for setting future injury prevention priorities.

## Methods

This is a prospective descriptive study in which injury data were collected over 26 weeks of the CIC for two consecutive years (April 2006 to March 2008). During this period, 7726 recruits enlisted at the Infantry Training Centre. Each recruit was invited to take part in the study during the initial medical assessment. The Ethics Committee of Teesside University approved the study and informed consent was provided by all study participants. A total of 1118 (14%) recruits were excluded from the study because: they did not provide consent; they failed the initial medical assessment; they were transferred to another regiment with no reported injury; or they took voluntary exit from training with no reported injury. In total, 6608 recruits were included in the study and were fairly homogeneous in terms of age (18.9 ± 2.3 y), height (176.5 ± 7.8 cm), mass (69.2 ± 9.7 kg) and body mass index (22.1 ± 2.5 kg/m^2^). The recruits were from the Line (66.2%), Guards (15.8%), Parachute (11.1%) and Gurkha (6.9%) Regiments. The training is longer for the Parachute (plus two weeks) and Gurkha (plus 10 weeks) Regiments, but injury data were only collected up to 26 weeks to ensure similar exposure to training across the Regiments.

At the beginning of military service all Infantry recruits undertake the CIC, which is divided into Phase I (weeks 1–13) and Phase II (weeks 14–26). On average recruits undertake 22 hours of military training per week with a gradual increase in load. The training demands vary slightly between Regiments, but energy expenditures typically exceed 5000 kcal per day [1]. The training programme includes lessons on military skills and a structured and progressive physical fitness programme. The training is standardised across military units and has been validated by the Army Recruiting and Training Division.

Recruits with a suspected injury reported to the medical centre within the camp and were seen by a physician for assessment and diagnosis. Musculoskeletal injuries were defined as pain, inflammation or a functional disorder that involved the bones, joints, muscles, tendons, ligaments, and associated connective tissue injury [5,16,20]. Where necessary, X-ray and/or MRI scans were used to confirm or reject initial clinical diagnoses [21]. Recruits with a diagnosed musculoskeletal injury were referred to the Physiotherapy Department for treatment. Blistering or cellulitis were not included in the categorisation of musculoskeletal injuries [5] as these injuries did not require rehabilitation. Musculoskeletal injuries with no definitive diagnosis were classified as ‘other’ [20].

The treatment programmes are designed to protect the injury and promote healing. Treatment for musculoskeletal injuries includes soft tissue and joint mobilisation, stretching, electrotherapy, acupuncture, exercises and relative rest followed by a graduated return to fitness [22,23]. Biomechanical and muscle imbalances, and functional strengthening and cardiovascular training are also addressed. Management of these injuries is guided primarily by the recruits’ conditions, symptoms and their response to treatment [24]. In some cases the injury can be managed with a reduction in the training load and volume, whereas in more extreme cases the recruit is removed from training altogether. The ITC adopts three phases of rehabilitation: pre-recovery, recovery and mainstream based on the severity of the injury. All treatment programmes in this study were supervised by a remedial exercise instructor and a physiotherapist. The pre-recovery phase involves non-impact activities that mainly focus on maintaining cardio-respiratory fitness such as swimming, balance, stretching and strengthening. The recoveryphase involves progressively longer and more challenging balance and proprioception exercises, progressive strengthening exercises with injured and non-injured limbs and jogging/running. The mainstream phase involves the recruit returning to a pre-injured level of activity including running, loaded marching and military-specific activities in order to prepare recruits for a full return to training. The successful completion of functional tests enables progression to the next phase of rehabilitation. The recruits spent up to 4 hours per day, five days a week in rehabilitation, depending on their stage of recovery. For the remaining time, recruits are involved in military training which is tailored to the phase of recovery. Rehabilitation time (days) for each individual case was determined by the number of days between arrival and discharge (last appointment) from the rehabilitation clinic. Accumulated rehabilitation times (days) for each injury specific diagnosis were recorded.

**Figure 1:**
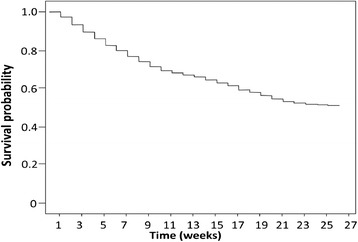
Kaplan-Meier survival curve for the proportion of survival of musculoskeletal injury during 26 week of training periods.

The survival probabilities for all musculoskeletal injuries were calculated (Figure 1) using a Kaplan-Meier analysis [25,17]. Exposure time was defined as the length of time recruits spent in training prior to injury [25]. The incidence of musculoskeletal injuries was the number of injury events expressed as a percentage of participants monitored. A chi-square test was used to examine differences in the frequency of injuries between Phase I (weeks 1–13) and Phase II (weeks 14–26) of training. Confidence intervals (95%) for injury specific diagnosis incidence, and means and standard deviations for rehabilitation times, were also calculated. The accumulated rehabilitation times for injury specific diagnosis are expressed as a percentage of the total number of rehabilitation days for all injuries. Statistical analysis was undertaken using Statistical Package for the Social Sciences v18.0 for Windows software (SPSS Inc., Chicago, IL).

## Results

The survival plot (Figure 1) shows the proportion of recruits who were free from injury at each week of the 26 week training. A higher number of injuries were reported in Phase I (n = 2242) than in Phase II (n = 984; p < 0.01) of training, with the highest rate of injury occurring in week two. Overall, almost half the recruits (48.65%) sustained at least one musculoskeletal injury during 26 weeks of CIC training.

**Figure 2:**
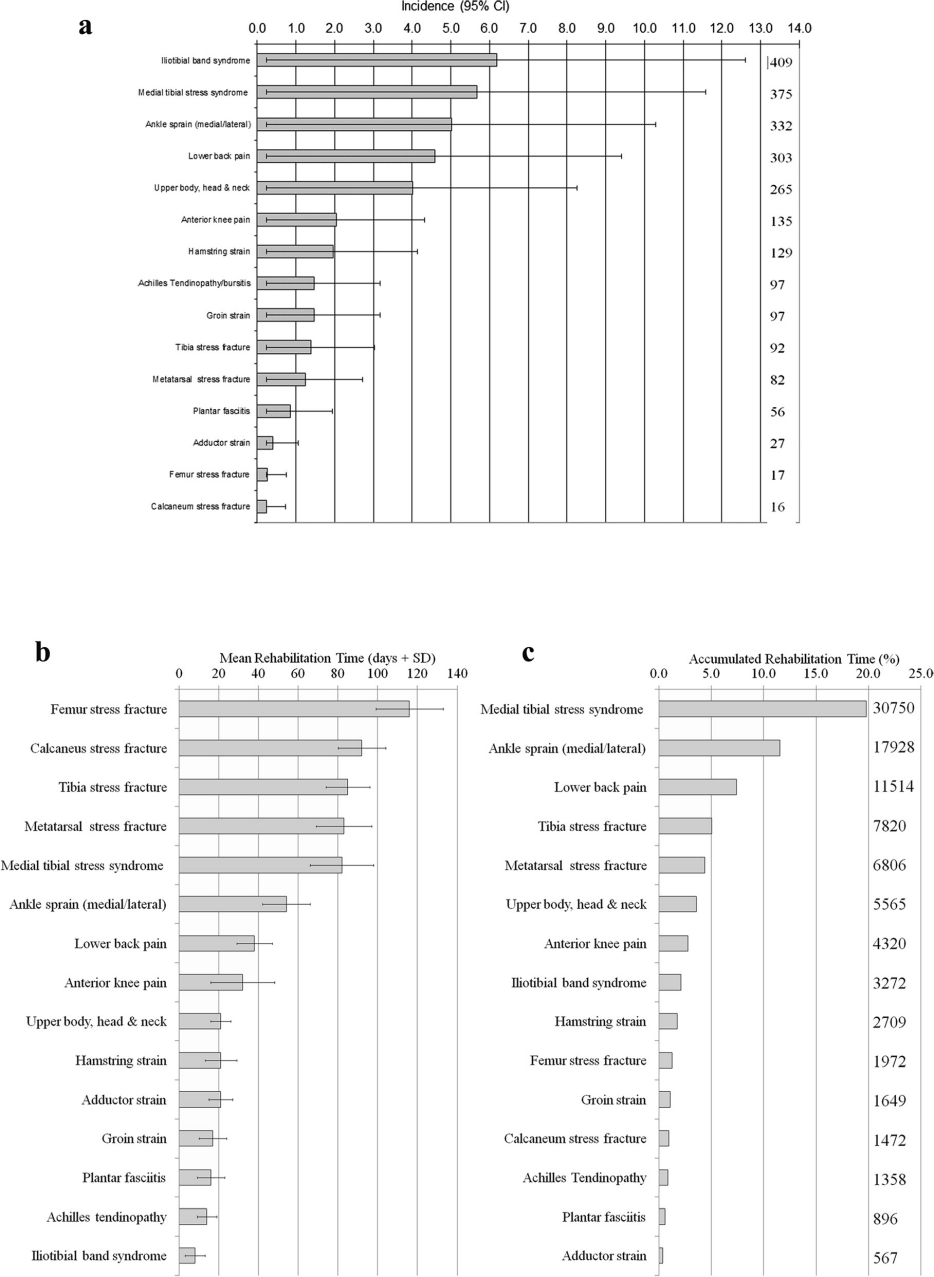
Musculoskeletal injuries in a cohort of 6608 recruits during a 26-week period of initial military training in the British Army. Data were recorded over a two-year period. **a)** The incidence of injuriesspecificdiagnosis expressed as percentage of 6608 recruits and 95% Confidence Interval . In the inset is the total number of injury events. **b)** Descriptive statistics of rehabilitation time (mean ± 1SD) for diagnosis-specific musculoskeletal injuries. **c)** Accumulated rehabilitation time for diagnosis-specific injuries expressed as percentage of all rehabilitation days. In the inset is the total number of days of rehabilitation for injury diagnosis.

The five most common diagnoses (Figure 2a) were iliotibial band syndrome (6.19%, 95% CI: 5.96-6.42), medial tibial stress syndrome (5.67%, 95% CI:5.44-5.91), ankle sprains (5.02%, 95% CI:4.78-5.26), lower back pain (4.59%, 95% CI:4.35-4.82) and combined upper body, head and neck (4.01%, 95% CI:3.77-4.25). Rehabilitation times (Figure 2b) ranged from 8 ± 5 days for iliotibial band syndrome to 116 ± 17 days for femoral stress fractures. Stress fractures typically took over 80 days to rehabilitate back into training. A total of 155,403 rehabilitation sessions took place within the clinic during the course of the study. The injuries having the greatest impact (Figure 2c) in terms of recovery days (number of days between the first and last rehabilitation session) were medial tibial stress syndrome (19.8% of total recovery days), followed by ankle sprains (11.5%), lower back pain (7.4%) and tibia stress fracture (5.0%).

## Discussion

Despite the widespread problem of musculoskeletal injuries in military recruits there is no proven single effective intervention for reducing all injuries [16]. There is, however, emerging evidence that interventions focusing on specific injuries can be effective in reducing the incidence of injury during initial military training [17,18]. In this study, injury records from a large cohort of military recruits were followed to provide an objective measure of impact of specific injury diagnoses during an initial 26 week military training programme. It is expected that information on the impact of specific injury diagnoses could be useful in facilitating a targeted approach to injury management.

There have been many studies reporting injury data for military populations and these, for the most part, focus on the incidence of injury. The overall incidence of injury in the current study (48.6%) is comparable with those values reported for other military populations [5,11,20-26] and for civilian runners [27]. It is, however, lower than in professional dancers [28] and other military-based studies [12] possibly due, in part, to our exclusion of blistering, variable physical demands of training and/or different demographics of the study populations. The timing of these injuries appears to be consistent with previous studies, with higher incidences occurring in the early weeks of the training programme [5,16]. In addition, the incidences of injury specific diagnoses are similar to previous studies of military populations [5]. For example, in this study the incidence of iliotibial band syndrome (6.2%) is similar to that reported (5.3%) by Almeida [5]. Similarly, the incidence of medial tibial stress syndrome (5.7%) is comparable with previous studies from civilian populations [29] and previous cohorts at ITC [30]. These trends may have relevance to other military and some athletic populations.

Although it is more difficult to compare the recovery times of musculoskeletal injuries with previous studies as these data are less frequently reported, there are again similarities between this and some previous studies. For example, the rehabilitation times for stress fractures of the femur, calcaneum and tibia in our centre were 116, 92 and 85 days, respectively, compared with an average time of 99 days (range 14 to 672 days) [31] and, specifically, 101 days (range 56 to 335 days) for the neck of femur [24,32,33], with tarsal stress fractures taking the longest time to recover [31,34]. However, before suggesting that our observations may translate to other populations, we also found much shorter rehabilitation times for other soft tissue injuries. For example, a typical case of Achilles tendinopathy takes only 14 days to recover in our centre whereas this recovery time in a different population can be as high as several months depending on the stage of the pathology, age, duration of symptoms and occurrence of tendinopathic changes [35-37]. Mild reactive stage cases of Achilles tendinopathy can heal in just a few weeks while chronic tendinopathy recovery can be a lengthy process that may take several months [37]. Obtaining medical attention at an early stage may improve recovery outcome, as treatment becomes more complicated and less predictable when the condition becomes chronic and may require surgical treatment [36,37].

Musculoskeletal injuries are generally considered to be the result of large, frequent and localised internal stresses in the body. The loading conditions created during marching with and without external loads (backpack), and other general military training will determine the nature of these stresses. There are potential strategies that could be adopted to manage these internal stresses more effectively, which may be useful when planning an injury prevention and/or rehabilitation programme [5,16]. First, it may be possible to reduce the sudden and abrupt load exerted on the recruits. We observed many more occurrences of injury in the early weeks of the training programme. While the musculoskeletal system will adapt to a new intensive physical regime by neuromuscular strengthening or remodelling, this process of adaptation requires time. Unfortunately, the physical demands during the CIC [38] are highest in the first 9 weeks of training, reaching a peak of physiological stress in week two, suggesting a mismatch between training loads and the ability of the recruit to cope with the internal stresses. This mismatch, in combination with the additional psychological stress of the new environment [39,40], is expected to contribute to the high incidence of injuries in these early weeks.

Another potential strategy would be to select recruits who are already able to cope with such demands. The British Army already adopts a screening process but at present this is focused on aerobic fitness, physical strength and trainability. In short, recruits who pass the screening process may be predisposed to injury. It is noteworthy that many athletic programmes screen an athlete on the basis of their ‘quality of movement’. These subtle measures are believed to provide an insight into the athlete’s robustness and could potentially be used alongside traditional measures of fitness and trainability at recruitment. A third strategy would be to modify the way in which the recruits walk using gait retraining and/or foot orthoses. Gait retraining, a combination of exercises to improve neuromuscular control and bio-feedback to reduce known biomechanical risk factors, has been shown to reduce loads on the tibia [41]. More recently, this approach has led to a reduction in the incidence of injury [18] as have well-designed foot orthoses [9,42]. In addition to prevention, there may scope to accelerate the process of rehabilitation. The current care pathway for many overuse injuries includes rest with partial weight-bearing using crutches with some activity modification until the pain subsides [43]. These prolonged periods away from training are known to decrease motivation [13] and these conservative approaches show a poor response for many overuse injuries [44]. Further work on improving rehabilitation through a combination of exercise, nutrition and therapy is warranted.

Overall, it is clear that incidence and recovery times should be considered simultaneously in order to prioritize and develop an injury management programme. Based on the incidence data alone, some injuries such as iliotibial band syndrome have alarmingly high incidences. However, these injuries are shown to be relatively easy to rehabilitate [45], resulting in a low accumulated rehabilitation time. Likewise, stress fracture of the femur takes a long time to recover yet the incidence and the accumulated impact of this injury remain low. In contrast, ankle sprains, lower back pain and stress fractures to the tibia/metatarsals occur frequently and usually need substantial rehabilitation, and will benefit from targeted interventions. Medial tibial stress syndrome was, by far, the most significant injury. This injury has a high incidence and a long rehabilitation time and although we did not include the costs of injury specific diagnosis (*eg* consumables) by health economic modeling, this injury accounted for almost one-fifth of all recovery days. This type of injury is in need of urgent and proactive management for prevention, rehabilitation or both, and a re-examination of current practice may be justified.

The main strength of this study is that it provides new meaningful data from a large cohort of recruits who are training in a controlled environment. The data will provide valuable baseline evidence to the scale of the injury problem and will contribute to developing a systematic approach to planning and priority-setting in injury management [46]. An additional strength is that the diagnoses were made by experienced medical professionals working in an Army medical environment. It is also noteworthy that the participation rate was high (>85%) and the duration of data collection allowed us to capture injury events over several training cycles. There are, however, some limitations to this study. First we did not include health economics in this study and the impact of injury on lost training days, medical support costs, and reduction in operational readiness would require formal evaluation in future. However, it is reasonable to suggest that manning the clinic represents a substantial proportion of these costs. Thus, the accumulated rehabilitation times presented in this study are considered to serve as a useful and pragmatic proxy measure for economic costs. Second, our cohort is predominantly male and homogeneous with respect to age, height and body mass. It is, therefore, very difficult to predict how these findings might translate to other institutes and populations. While the incidence of specific injuries may not be broadly generalizable, the rehabilitation times provide important references to other young physically active male populations. Third, we did not monitor training load in these participants and thus we are unable to quantify the relationship between load exposure and injury risk. The relationship is not expected to be linear [47], but further work would enable more effective strategies to be developed for controlling these harmful stresses.

## Conclusions

This study provides a baseline framework for setting strategies to improve and prioritise injury prevention and rehabilitation in the British Army, and may have implications for other military and young male athletic populations.

## Abbreviations

CIC: Combat Infantryman’s Course
